# N‐P utilization of *Acer mono* leaves at different life history stages across altitudinal gradients

**DOI:** 10.1002/ece3.5945

**Published:** 2019-12-18

**Authors:** Zhaopeng Song, Yanhong Liu, Hongxin Su, Jihua Hou

**Affiliations:** ^1^ Key Laboratory for Forest Resources & Ecosystem Processes Beijing Forestry University Beijing China; ^2^ Key Laboratory of Beibu Gulf Environment Change and Resources Utilization of Ministry of Education Nanning Normal University Nanning China

**Keywords:** *Acer mono* Maxim, allometric growth, altitude gradients, life history stage, N‐P stoichiometric

## Abstract

The relationship between plants and the environment is a core area of research in ecology. Owing to differences in plant sensitivity to the environment at different life history stages, the adaptive strategies of plants are a cumulative result of both their life history and environment. Previous research on plant adaptation strategies has focused on adult plants, neglecting saplings or seedlings, which are more sensitive to the environment and largely affect the growth strategy of subsequent life stages. We compared leaf N and P stoichiometric traits of the seedlings, saplings, and adult trees of *Acer mono* Maxim and different altitudes and found significant linear trends for both life history stages and altitude. Leaf N and P content by unit mass were greatly affected by environmental change, and the leaf N and P content by unit area varied greatly by life history stage. *Acer mono* leaf N‐P utilization showed a significant allometric growth trend in all life history stages and at low altitudes. The adult stage had higher N‐use efficiency than the seedling stage and exhibited an isometric growth trend at high altitudes. The N‐P utilization strategies of *A. mono* leaves are affected by changing environmental conditions, but their response is further dependent upon the life history stage of the plant. Thus, this study provides novel insights into the nutrient use strategies of *A. mono* and how they respond to the environmental temperature, soil moisture content along altitude and how these changes differ among different life history stages, which further provide the scientific basis for the study of plant nutrient utilization strategy on regional scale.

## Graphical abstract

The researchers investigated leaf nutrient utilization strategies during the seedling, sapling, and adult stages of *A. mono* under different moisture and temperature conditions.

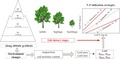

## 1 INTRODUCTION

The relationship between plants and the environment is a core area of research in ecology (Molina‐Venegas, Aparicio, Lavergne, & Arroyo, 2018; Siefert, Fridley, & Ritchie, 2014). Plant growth is affected by different environmental factors, which can be reflected by variation in plant functional traits (Huang, Pedersen, Fredriksson, & Thygesen, 2019; Wei, Savage, Riggs, & Cavender‐Bares, 2017). Leaves are the photosynthetic organs of plants, and as such are involved in the circulation of matter and the flow of energy through an ecosystem. Thus, leaf functional traits can be considered to represent the response of a plant to environmental factors (Onoda et al., 2017; Shi, Liu, et al., 2019; Shi et al., 2018; Song & Liu, 2019; Su et al., 2019). Plant N and P are important elements that influence plant metabolism, energy transmission, and ecological processes (Abdalaroberts et al., 2018). Leaves usually have a relatively stable stoichiometric composition over long‐term environmental adaptation, and their stoichiometric characteristics can scale the adaptation mechanism from the plant organ to the community level and even regional level (Hou et al., 2018). Further investigating leaf stoichiometry under different environmental conditions could provide insights into the relationship between regional biogeochemical cycles and vegetation (Funk et al., 2017; Martin & Isaac, 2018). Studying the response of leaf stoichiometry to environmental factors at the regional scale is important for revealing the underlying adaptation mechanisms in plants (Sistla & Schimel, 2012). This can provide a fuller understanding of the mechanisms of regional‐scale vegetation response to environmental changes, and predictions for the response of plants to habitat changes under global climate change scenarios.

Vegetation differentiation at the altitude gradients under environmental influences is an important part of plant response to global changes (Zhang et al., 2017). Changes in rainfall and temperature along altitude gradients exhibit similar patterns to changes along latitude gradients (Achat, Augusto, Gallet‐Budynek, & Loustau, 2016). Meanwhile, soil moisture content is directly related to rainfall (Molina‐Venegas et al., 2018). Plant growth and development varies along altitude gradients owing to differences in light exposure, temperature, and precipitation along these gradients (Shi, Chen, Hui, & Grissino‐Mayer, 2016; Shi et al., 2017; Yin et al., 2018). The content of N and P in plant leaves is highly affected by environmental conditions such as soil nutrient content, growth season temperature, and annual precipitation. Thus, leaf stoichiometry undergoes complex adaptive changes according to the environmental changes along the altitude gradients (Tao et al.., 2010). Leaf N and P content were found to decrease with increasing latitude and decreasing temperature, and the N‐P stoichiometry of plants in arid areas is higher (Minucci, Miniat, Teskey, & Wurzburger, 2017). Some previous studies have reported that rainfall and temperature conditions became increased along the elevation gradient, the accumulation of humus increased, and the content of organic matter, total N, and total P in the soil increased with increasing altitude (Reich, Oleksyn, & Tilman, 2004). Therefore, soil moisture and temperature are affected by altitude, and further affects in the leaf stoichiometric characteristics.

The response of plants to the environment is dependent upon their stage of life development, which means that adaptation strategies may vary during different life history stages (Gustafsson et al., 2016). Previous study has found that as diameter at breast height (DBH), specific leaf weight, leaf N content, and water‐use efficiency also increased (Renninger, Carlo, Clark, & Schäfer, 2015). In recent years, more and more attention has been paid to the study of the allometric growth relationship of plant leaf stoichiometry (Leigh, Sevanto, Close, & Nicotra, 2017; Li, Zheng, Fan, Zhong, & Cheng, 2017). The analysis of these relationships is more informative than correlation analyses of the intrinsic structures and physiological changes of plants and allows for the analysis of nutrient use strategies during plant growth (Temme et al., 2017). Compared with the differences in leaf trait among the major floristic regions, the results suggest that the allometric scaling of leaf traits varied among these global floras (Heberling & Fridley, 2012). However, previous research on plant adaptation strategies has mostly focused on the adult stage and has neglected to note that seedlings are more sensitive to the environment and that the growth of seedlings determines the strategy of subsequent stages (An & Shangguan, 2008; Dutcă et al., 2018; Ishida, Yazaki, & Hoe, 2005). Meanwhile, allometric growth patterns in different life stages of a species can accurately reflect specific changes in leaf N:P stoichiometry (Bloomfield et al., 2018). However, the related research is seldom reported (Li et al., 2017; Savage et al., 2016). Considering the environmental heterogeneity along altitude gradients and the variable response of plants during different life history stages, it is also important to study the influence of the environment on plant growth strategies.

Based on the above studies, it is apparent that leaf N‐P stoichiometry and nutrient utilization strategies of plants differ at different altitudes. The changes in allometric growth rates under different habitat conditions reflect plant nutrient utilization strategies and can reveal how adaptable plants are to their environment (Dutcă et al., 2018). In the present study, to comprehensively investigate the effects of environmental factors on leaf N:P stoichiometry, environmental temperature and soil moisture content (SMC) were analyzed against leaf N and P content to determine how plants adapt their nutrient use strategies to environmental change. Our main objectives were to determine 1. the effects of changes in hydrothermal conditions along altitude gradients on the leaf N:P stoichiometry of plants during different life stages; 2. whether there is a consistent trend in the allometric growth of leaf N‐P stoichiometry at different stages of life history; and 3. whether the allometric growth changes under different environmental conditions during different life history stages are reflected in differences in nutrient utilization strategies of plants.

## 2 MATERIALS AND METHODS

### 2.1 Study site

**Figure 1:**
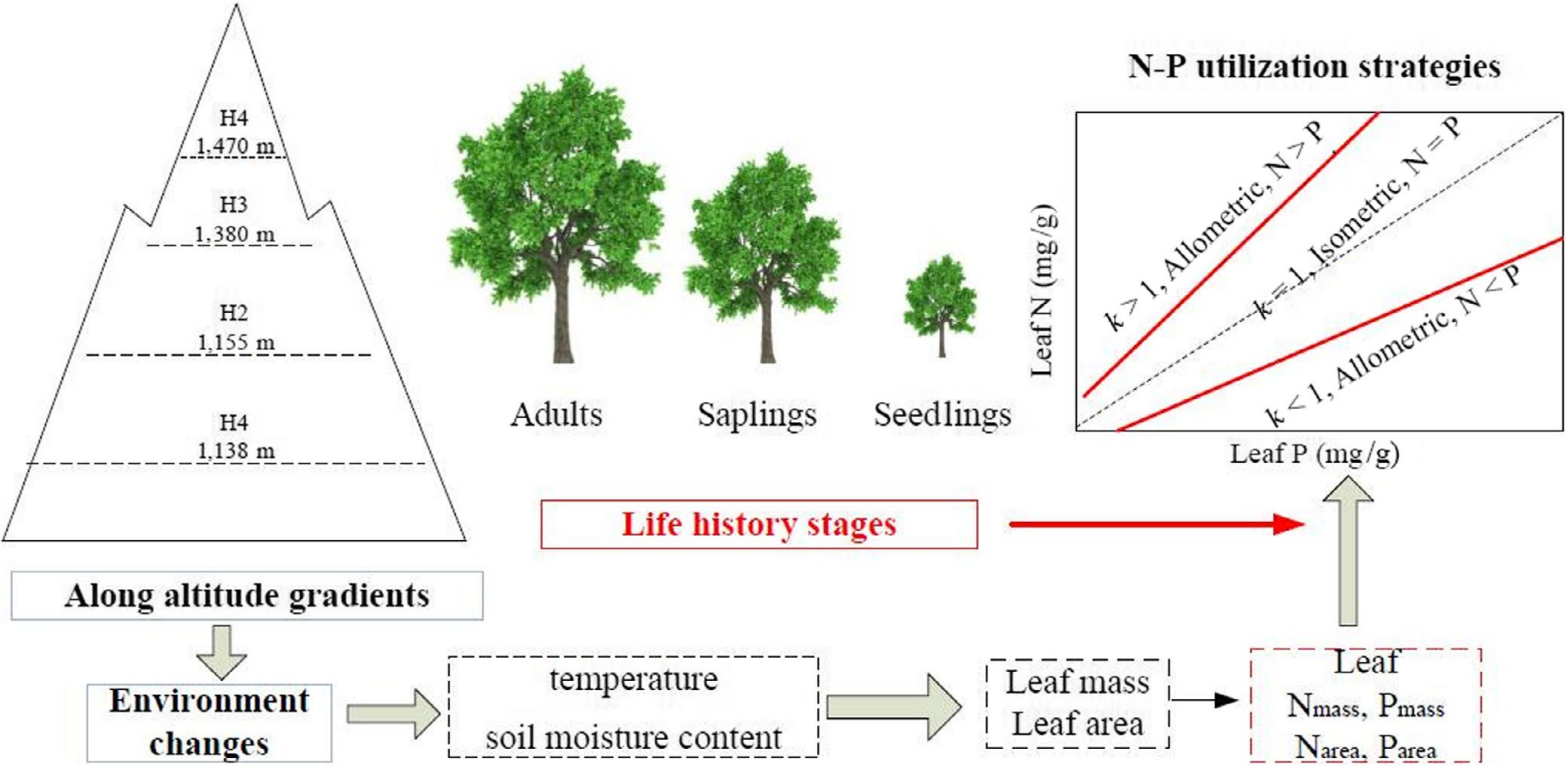
Graphical figure of main research

The study site was in the Dongling Mountains in China, which play an important role in biodiversity protection and water conservation in the Beijing area (Sadia et al., 2017). The mountains located in the Beijing Forest Ecosystem Research Station (40°00′N, 115°26′E), and all the climatic data form this research station. The region has a temperate continental monsoon climate with a mean annual temperature of 4.8°C (January 10.1°C; July 18.3°C). The annual precipitation is about 612 mm, 78% of which occurs from June–August. As an important topography of the west of Beijing, Dongling Mountain is a suitable site for research under the environmental conditions along the vertical latitudes, and its deciduous broad‐leaved forest group is of typical significance to the forest in the warm temperate zone of North China. Its zonal vegetation type is warm temperate forests of *Quercus liaotungensis* Mayr., *Betula platyphylla* Suk., *Betula dahurica* Pallas, *Juglans mandshurica* Maxim., and *Populus davidiana* Dode (Fang, Liu, Zhu, Wang, & Liu, 2007). *Acer mono*, widely distributed along the altitudinal gradient in this area, is an important common tree species in these forests, an ideal study species. In the present study, we investigated leaf nutrient utilization strategies during the seedling, sapling, and adult stages of *A. mono* under different moisture and temperature conditions (Figure 1).

The field sampling was carried out in July and August 2017. Four 30 m × 20 m plots were established at 20‐m intervals in each of the four altitude gradients: 1,138 m (H1), 1,155 m (H2), 1,380 m (H3), and 1,470 m (H4), totally distributed the sixteen plots (four altitude gradients × four repeats) (Figure 1). The latitude, longitude, and altitude of each sample plot were recorded by GPS. The soil moisture content was measured by collecting fresh soil samples and drying them at 105°C for 24 hr to constant weight, and calculating the difference before and after calculation. The temperature was calculated by the rise of altitude based the mean annual temperature (Table 1).We also recorded community species composition, DBH and height of all adult trees of *A. mono*, basal diameter of *A. mono* saplings and seedlings. At each development stage of *A. mono*, we recorded at least 50 individuals. At each altitude, 15 *A. mono* adult (DBH about 40 cm), 15 saplings (height about 2 m), and 15 seedlings (height 0.3–0.5 m) sample were subsampled from each 50 individuals. After visual inspection, we sampled 20 leaves without pests and diseases from each selected individual by using high‐branch shears. We selected fully expanded leaves located in the middle to outer layers of the crown. The leaves were stored in self‐sealed bags in a portable refrigerator for transportation to keep them fresh until we were able to dry them.

**Table 1:** Characteristics of environment and community under four vertical altitude gradients

Abbreviation	Altitude(m)	SMC and T/H		Dominant and common species
		Temperature(°C)/*T*(°C)	Soil moisture content (%)/SMC (%)	
H1	1,138	5	51.29	*Juglans mandshurica*; *A. mono*; *Fraxinus Americana*; *Pyrus ussuriensis*; *Rhamnus parvifolia*
H2	1,155	4.9	29.24	*Populus cathayana*; *A. mono*; *P. ussuriensis*; *J. mandshurica*
H3	1,380	3.6	13.39	*Quercus wutaishanica*; *B. platyphylla*; *B. dahurica*; *A. mono*; *F. americana*
H4	1,470	3	36.05	*B. platyphylla*; *P. davidiana*; *A. mono*; *B. dahurica*; *Sorbus discolor*

Note

H1, H2, H3, and H4 represent abbreviations of the four altitude gradients, respectively.

Abbreviations: H, soil moisture content and temperature conditions; SMC, soil moisture content; *T*, temperature.

### 2.2 Chemical analyses

The area, dry mass, and N and P concentration were measured for each of the sampled leaves. The leaves were scanned using HP Scanjet G3110 (HP), and the pictures were analyzed by using MapInfo12.5 (MapInfo^®^ software, 2017), and leaf area was recorded. Leaves were oven‐dried to a constant mass at 65°C for 24 hr and then weighed. The average leaf mass and leaf area were calculated as the total mass or area divided by leaf number. Dried leaves were digested by H_2_SO_4_‐H_2_O_2_. The total N concentration of leaves was determined by dry combustion using Vario MAX CN Elemental Analyzer (Elementar). Leaf P concentration was measured by the Mo‐Sb colorimetric method using Hitachi U‐3900 (Hitachi).

Leaf N content per unit mass (N_mass_, mg/g) was calculated as total N content (mg)/dry weight (g).

Leaf N content per unit area (N_area_, mg/ cm^2^) was calculated as N_mass_ (mg/g) × dry weight (g)/ area (cm^2^).

Leaf P content per unit mass (P_mass_, mg/g) was calculated as total P content (mg)/dry weight (g).

Leaf P content per unit area (P_area_, mg/ cm^2^) was calculated as P_mass_ (mg/g) × dry weight (g)/area (cm^2^).

### 2.3 Data analysis

Differences in the leaf N and P content and the N:P ratios among individuals composite sample of leaves from certain plots plant leaves at different life stages and altitude gradients were tested using a one‐way analysis of variance (ANOVA) with Duncan's post hoc tests. Linear regression was employed to explore the trends in N and P content and N:P ratios of individuals in each life history stage along the altitude gradient. The effects of soil moisture content and temperature conditions (H) and life history stages on the variation of leaf stoichiometry were further quantified by general linear models (GLMs) and partial GLMs. To avoid collinearity among explanatory variables, correlated predictors were removed by multiple stepwise regression (*p* < .05).

The scaling relationship of N and P in leaves was described by the equation *Y = *α*X* ^β^ or its log‐transformed form log(*Y*) = log (α) + β log(*X*), where *X* and *Y* are the P and N content of leaves of individuals at different life stages, respectively, log (α) is the intercept on the y‐axis that is actually a relationship to be fitted, and β is the slope of the linear equation, representing the allometric exponent.

The data for leaf N content and leaf P content were log‐transformed. Reduced major axis (RMA, also called standardized major axis) regression was used to determine the scaling exponent and constant of log–log linear functions (Warton, Wright, Falster, & Westoby, 2010). Confidence intervals of the slope were calculated according to the method of Pitman (Pitman, 1939). A likelihood ratio test (Bartlett's amendment) was used to test the heterogeneity of the slope of leaf traits at different life stages (Warton & Weber, 2015). If there was heterogeneity in the slopes, multiple post hoc comparisons between the slopes were performed. If there was no significant difference between the slopes, a common slope would be given and the intercept difference and the displacement along the common principal axis were examined. The displacement of *A. mono* leaf traits along the common major axis at different stages of life history indicates differences in leaf stoichiometry among these stages. Differences in the regression slopes among different life stages were tested by multiple post hoc comparisons. The significance level for testing slope heterogeneity and differences from slope = 1 was *p* < .05. All statistical analyses were performed using R software (version 3.3.2, R Core Team 2017).

## 3 RESULTS

### 3.1 **Differences in leaf N‐P stoichiometry of *A*** *.* ***mono* during different life history stages at different altitudes**

**Figure 2:**
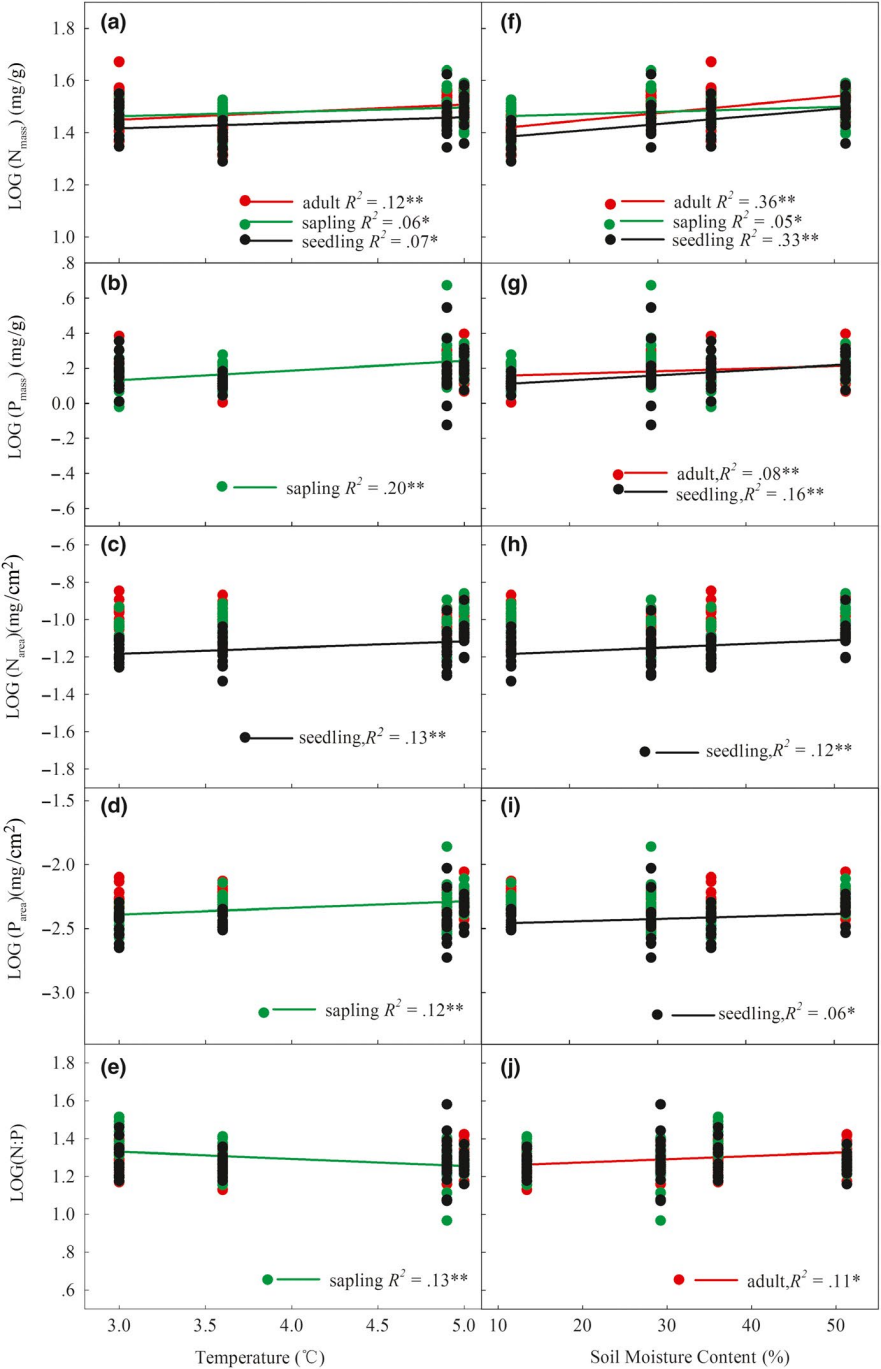
Changes in N and P stoichiometric of *A. mono* leaves with temperature and soil moisture content at different stages of life history. *: indicates significant (*p* < .05) linear regression; **: indicates extremely significant (*p* < .01) linear regression. LOG stands for logarithmic transformation of data

N_mass_, P_mass_, N_area_, and P_area_ increased significantly at higher temperatures and soil moisture content (SMC) (Table S1, *p* < .05). The linear trends of N_mass_, P_mass_, and P_area_ significantly increased with the increase of the temperature in saplings (Figure 2a,b,d,* p* < .05). N_mass_ and N_area_ have a significant linear relationship at the seedling stage (Figure 2a,c). N_mass_ had a significant linear relationship in the adult stage (Figure 2a), while leaf N:P ratio showed a significant downward linear trend in the sapling stage (Figure 2e). With increasing SMC, N_mass_, P_mass_, N_area_, and P_area_ significantly increased in the seedling stage (Figure 2f–i). N_mass_, P_mass_, and N:P increased linearly during the adult stage (Figure 2f–h), and N_mass_ also showed a significant linear increase during the sapling stage (Figure 2f). This suggests that the leaf N‐P stoichiometry of *A. mono* exhibited a regular linear trend in different life history stages when the environmental conditions changed.

The correlation and factor contribution analysis between N‐P stoichiometry characteristics of *A. mono* leaves and changes in life history stage and environmental factors were compared (Table 2). The relationship between leaf N‐P stoichiometry and life history stage and environmental conditions showed significant or extremely significant correlations (*p* < .05 or *p* < .01) (Table S2). Above analysis indicating that the N_mass_ and P_mass_ are greatly affected by environmental change and that the N_area_ and P_area_ are greatly affected by life history stage, which further provides a basis for study of the allometric rates in the environment.

**Table 2:** The contribution of factors affecting N and P content in the leaves of *A. mono*

Group	N_mass_ (%)	P_mass_ (%)	N_area_ (%)	P_area_ (%)	N:P
H	20.29	8.14	2.44	2.48	3.65
stage	6.16	0.26	35.22	12.13	0.83
H*stage	26.45	8.40	37.66	14.61	4.48

Note

H, soil moisture content and temperature conditions; N:P, N‐P ratio; N_area_, N content per unit area; N_mass_, N content per unit mass; P_area_, P content per unit area; P_mass_, P content per unit mass.

### 3.2 Response of N‐P stoichiometry utilization of *A. mono* leaves under different environment conditions and life history stages

The leaf N concentration was significantly correlated with the P concentration (Figure 2, Table 3). The common slope of N_mass_ and P_mass_ standard axis (SMA) was 0.73 (95% confidence intervals: CI = 0.65–0.83), which was significantly different from 1 (Table 3, *p* < .01). Similarly, the common slope of N_area_ and P_area_ SMA was 0.80 (95% confidence intervals: CI = 0.73–0.89), indicating a significant allometric relationship (*p* < .01). This shows that the demand for P was greater in the plant, indicating higher levels of P utilization.

**Table 3:** Standardized main axis (SMA) regression parameters of leaf N‐P stoichiometric ratios in *A. mono*

	Slope(CI)	R^2^	Heterogeneity of slopes (*p*)
P_mass_‐N_mass_	0.73 (0.65,0.83)	0.28**	<.01
P_area_‐N_area_	0.80 (0.73,0.89)	0.56**	<.01

** : indicates extremely significant (*p* < .01) linear regression between N and P. CI: indicates 95% confidence intervals. Heterogeneity of slopes were significantly different from 1 (*p* < .01).

**Figure 3:**
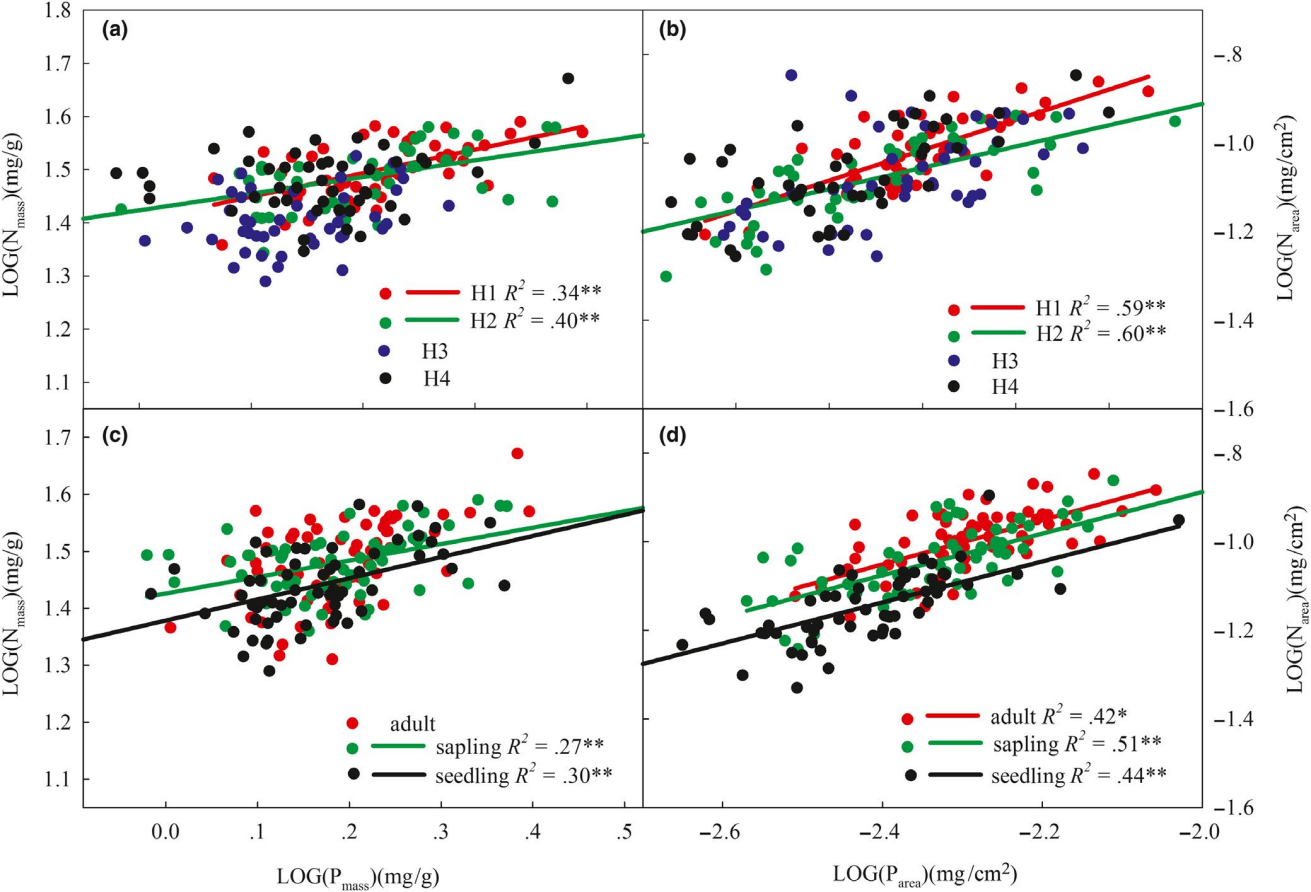
Allometric growth of *A. mono* N‐P under different environmental conditions and life history stages. H1, H2, H3, and H4 represent abbreviations of the four altitude gradients respectively; LOG stands for logarithmic transformation of data

Under H1 and H2, N_mass_‐P_mass_ and N_area_‐P_area_ of *A. mono* leaves exhibited extreme allometric growth, while isometric growth was observed at relatively low temperature plots at high altitudes(H3, H4) (Figure 3a,b, Table S3). Significant allometric growth patterns were found in the sapling and seedling stages, and they had significantly different slopes (Figure 3c, Table S3). The N_area_‐P_area_ showed significant allometric scaling in all stages, but there was no significant difference for the slopes among the three life history stages (Figure 3d, Table S3).

The shift along a common slope can indicate the rate of change for N and P content in *A. mono* leaves between different environmental factors (Figure 3, Table 4). At H2, the slope of N_mass_‐P_mass_ was the smallest, which indicated that *A. mono* invested significantly less N when per unit P mass increased at H2. Comparing the rate of change for N and P in different life history stages (Figure 3, Table 5) showed that, in the adult stage, the N_mass_ was significantly higher than that of the other life history stages with P and that in the sapling stage, N had the lowest utilization efficiency. The N_area_‐P_area_ exhibited a common slope at each life history stage. In addition, along with the change in the common slope, the larger intercept indicates a greater N content than P. The intercept of the adult stage increased significantly, indicating that adults can use more N per unit mass.

**Table 4:** Tests for heterogeneity of slope and shift in the intercept for leaf N‐P stoichiometric ratios of *A. mono* under different environmental conditions when the slopes were homogenous

Y	X	Shift along the common slope				Shift in elevation				Common slope not different from 1 (*p*)	Heterogeneity of slopes (*p*)
		H1	H2	H3	H4	H1	H2	H3	H4		
P_mass_	N_mass_	0.76a	0.49b	0.96a	0.75a	1.33a	1.39a	1.27b	1.36a	<.01	.003
P_area_	N_area_	0.89a	0.62b	1.03a	0.87a	1.03ab	0.38ab	1.33a	1.01b	<.01	.003

**Table 5:** Tests for heterogeneity of slope and shift in the intercept for leaf N‐P stoichiometric ratios of *A. mono* in different life history stages when the slopes were homogenous

Y	X	Shift along the common slope			Shift in elevation			H0 common slope not different from 1 (*p*)	Heterogeneity of slopes (*p*)
		Adult	Young	Seedling	Adult	Young	Seedling		
P_mass_	N_mass_	0.99a	0.55b	0.68b	1.30a	1.38a	1.33b	<.01	.01
P_area_	N_area_	0.77	0.66	0.70	0.77a	0.49b	0.54b	<.01	.52

### 3.3 Response of N‐P stoichiometry utilization of *A. mono* in different life history stages under different environment conditions

**Figure 4:**
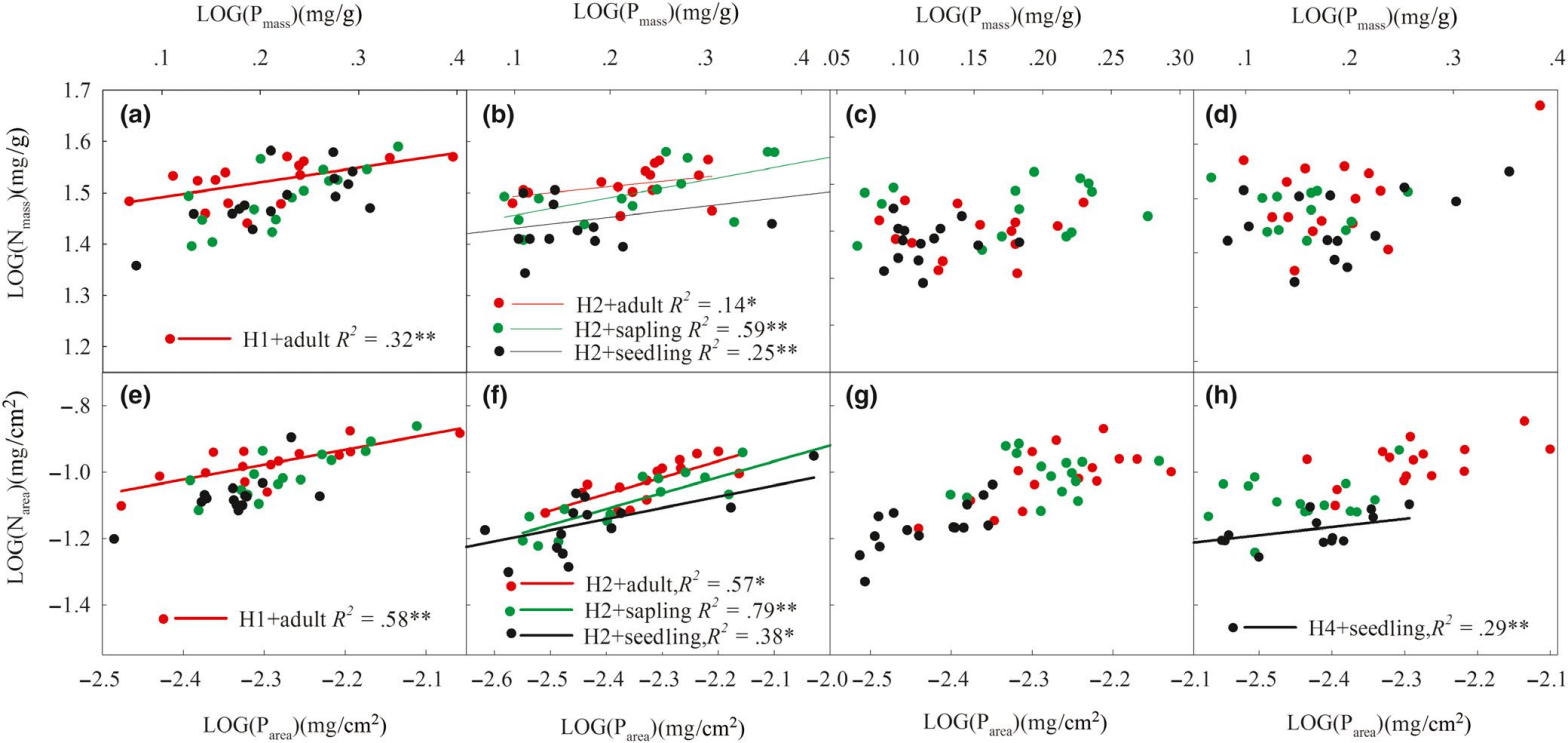
Allometric growth law of N and P utilization efficiency of *A. mono* at different growth stages under various environments. H1 + adult represent abbreviations of the adult life history stage under H1 altitude gradients, same as others; LOG stands for logarithmic transformation of data

For the stoichiometric relationships of *A. mono* at different growth stages in different environments, the N and P of adult *A. mono* showed a significant allometric growth trend in the H1 environment (Figure 4a,e, Table S4). The N‐P of *A. mono* at different stages of life history in H2 environment all showed a significant or extremely significant allometric relationship (Figure 4b,f, Table S4). The N_area_‐P_area_ of the *A. mono* leaf in the seedling stage showed a significant allometric growth trend in the H4 environment, and the N and P in other stages was all isometric growth (Figure 4d,h, Table S4).

## 4 DISCUSSION

### 4.1 Differences in life history stages and environment of N and P content in *A. mono* leaves

Certain types of terrain can form distinct microclimates, which indirectly affect the distribution of soil moisture and nutrients (Boutin et al., 2017). In temperate regions, environmental and ecological factors vary along altitude gradients and as altitude increases ambient temperature decreases, and annual precipitation, light exposure, and ultraviolet radiation increases, which affect the utilization of leaf nutrients (Wright et al., 2017). Photosynthetic capacity of leaves is related to N and P content, sustains life and reproduction and growth (Way, Stinziano, Berghoff, Oren, & Cernusak, 2017). In the present study, leaf N and P concentration was found to be significantly or highly significantly correlated with environmental factors and life history stages (Huang, Ratkowsky, et al., 2019). The N and P content of *A. mono* leaves responded differently to the changes in environmental factors along altitude gradients, and N_mass_, P_mass_, N_area_, and P_area_ increased significantly under higher ambient temperature and higher SMC, further indicating the significance of the linear relationships among them. The change in the environment under the altitude gradient is a microcosm of the environmental change along latitudinal gradient. Kerkhoff, Enquist, Elser, & Fagan (2005) considered that the N and P content of leaves decreases with increasing latitude. However, some previous studies have reported that on a large scale, the N and P concentration of leaves decreases with decreasing latitude and increasing annual average temperature.

Soil moisture content is mainly affected by precipitation, and higher soil moisture can promote the mineralization and nitrification of soil N (Jack brookshire, Gerber, Webster, Vose, & Swank, 2015; Mitchell, 2011), and the concentration of alkaline‐hydrolyzed N in the Dongling Mountain area is higher (Zhu et al., 2015). If the soil temperature is high, soil N mineralization is faster, and the available inorganic N content for plants is higher (Wilson & Jefferies, 1996), which provides more favorable soil conditions for plant growth, consistent with the results of our study. Reich et al. (2004) and Han, Fang, Guo, & Zhang (2005) reported similar results regarding grassland ecosystems in China scale, but they are not consistent with our results. The reason for this discrepancy is related to the scale of the experimental plots used and the species studied. The large‐scale study spans multiple latitude bands. In the selected experimental plot of the Dongling Mountain area, the main elevation areas of *A. mono* growth are concentrated in the range of 1,138 m to 1,470 m, with little vertical gradient span. Although temperature response has a similar trend to that of latitude, the effects of precipitation and SMC must also be considered (Reich et al., 2004). In addition, the environmental changes caused changes in the nutrient utilization strategy of *A. mono* through changing the dry weight and area of leaves, and thus have an impact on unit mass and unit area of leaf N‐P content (Huang, Ratkowsky, et al., 2019; Huang, Su, et al., 2019; Onoda et al., 2017). Our results also indicated that the N and P content per unit mass are greatly affected by environmental changes.

In our study, the N‐P stoichiometry of *A. mono* leaves showed consistent changes through life history stages, the N_mass_, N_area_, and P_area_ all significantly decreased in the seedling stage compared with the adult stage. Other studies also shown that the N and P content of plant leaves changed through the different life history stages (Li et al., 2014; Sterner & Elser, 2002). Besides, the per unit area N and P concentration of *A. mono* leaves was greatly influenced by the life history stage. Previous research indicated that plants at the seedling stage use more nutrients for photosynthesis, and the seedling stage invests more in the leaf area, and the area‐based nutrient content decreases (Brown, Gillooly, Allen, Savage, & West, 2004). As the plant grows and metabolizes, it requires more N to meet its own photosynthetic and growth demands, and thus photosynthetic capacity is increased accordingly; it needs a large amount of the necessary protein and nucleic acid, which increases the N and P content to meet the growth requirements (Zechmeister‐Boltenstern et al., 2016). At the same time, studies by Nabeshima & Hiura (2004) on *A. mono* showed that the mass‐based photosynthetic capacity decreased with the increase of life history stages leading to the concentration of N_mass_ and P_mass_ increasing.

### 4.2 The N‐P utilization strategy of *A. mono* leaves shown significantly changes in different life history stages and environmental conditions

The allometric relationship between N and P is supported by numerous previous studies. For example, at the regional scale, the N‐P allometric ratio between different sites, different functional groups, and different biomes shows significant differences (Hu et al., 2018; Reich, 2014; Reich et al., 2010). In our study, the N_mass_ and P_mass_ of *A. mono* showed a significant allometric growth trend, and the slope of the SMA of N_mass_ and P_mass_ was 0.73. Similarly, leaf N_area_ showed a significant allometric relationship with P_area_. The common slope of SMA was 0.80, indicating that the leaf N content is less utilization than the leaf P content. It was found that leaf N content was significantly related to photosynthetic capacity because N is an important constituent of an enzyme that drives photosynthesis (Vitousek, Porder, Houlton, & Chadwick, 2010). As a "loader" for protein synthesis, P is an important component of ribosomal RNA. More rRNA is required to participate in the synthesis of proteins to meet metabolic demands during plant growth, which results in the growth rate of P being faster than N (Bloomfield, Farquhar, & Lloyd, 2014).

Plants will invest more biomass for assimilation and support structures, as well as leaf nutrient use efficiency effected by leaf dry weight and leaf area and further effect the N_mass_, N_area_, P_mass_, and P_area_ (Franklin et al., 2017; Niklas et al., 2007; Shi, Li, et al., 2019). At the medium altitude environment (H2), the utilization efficiency of N_area_ in *A. mono* leaves was significantly lower than that at other altitudes with the increase of P. Previous studies have reported that the absorption efficiency of leaf N is the lowest at middle and low altitudes environment and that increasing altitude promotes the absorption of P by spruce leaves (Atkin et al., 2015). In our study, the N and P content per unit area and unit mass showed the same trend. At lower altitudes (H1 and H2), N‐P utilization showed a significant allometric growth. This indicated that under higher temperature and soil moisture conditions, the absorption of P from leaves was higher than that of N. Interestingly, N‐P utilization showed a constant velocity at high altitudes and relatively low temperatures. The soil moisture determines the development and evolution of soils, vegetation, and communities in high altitude, and further effect the water–heat balance of the entire ecosystem (Reich et al., 2004). Therefore, soil moisture content is one of the major limiting factors affecting plant growth and development (Rousseau et al., 2018). The N‐P utilization of *Q. liaotungensis* may also occur at constant velocity, because more water is used to protect or increase the density of mesophyll cells and reduce water loss when there is higher water availability at higher altitudes environment, resulting in an increase in the N content per unit leaf mass (Sakschewski et al., 2015; Zhang et al., 2018). At the same time, the lower temperature makes the growth season of plants relatively short and less N is allocated to the photosynthetic apparatus. Besides, photosynthetic capacity and net growth are lower, cell volume decreases, cell wall thickens, and tissue density increases, which increases per unit mass (Körner et al., 2016; Li, Kräuchi, & Dobbertin, 2006). Therefore, there will be isometric growth of N and P utilization at high altitudes.

The allometric relationships of N‐P utilization at different stages of life history were significantly different, which reflected the difference in N‐P utilization strategy during the growth of *A. mono*. Comparing the changes in N‐P utilization along the common slope in each stage, the N content in adult plants was significantly higher than that in the other life stages, and *A. mono* can therefore use more N in the adult stage. Li et al reported that the relationship between leaf area and leaf dry weight changed through the different stages of life history, and the difference in allometric relationships between saplings and trees reflected their resource utilization (Li et al., 2006). Differences in functional traits can affect stoichiometry adaptation strategies. Seedlings and saplings mainly live in an environment with insufficient light exposure under the forest canopy. In these stages, the rapid growth of leaves requires more P. Therefore, there is more demand for P, showing a trend of allometric growth in the use of P. Light conditions are sufficient, and the larger blades will reduce the exchange of photosynthetic gas, and the leaves need more chlorophyll to absorb sunlight and carry out photosynthesis to maintain growth at the adult stage (Savage et al., 2016). At the same time, growing trees need more N for mechanical support structures (Dean, 2004). Therefore, this highlights the difference between the growing stages and the adult stage.

### 4.3 Under different environmental conditions, the N‐P utilization shown significantly changes in different life history stages

Nowadays, more and more attention has been paid to the study of the allometric growth relationship of plant leaf stoichiometry (Leigh et al., 2017; Li et al., 2017). Previous studies have found that as diameter at breast height (DBH), specific leaf weight, leaf N content, and water‐use efficiency also increased, the photosynthetic N‐use efficiency and stomatal conductance decreased (Renninger et al., 2015). Notably, when *A. mono* adults were in the H1 environment, they exhibited a significant allometric growth trend, indicating that the higher temperature and soil moisture conditions allowed *A. mono* to maintain a rapid growth rate in the adult stage. However, H2 appeared to be the optimum environment for the growth of *A. mono* because a significant allometric growth trend was observed in all stages, showing rapid utilization of P for promoting growth. In the middle altitude areas (H2), soil moisture and temperature conditions are better, and the accumulation of humus increases, resulting in an increase in organic matter, total N, and total P in the soil (Wright et al., 2017). At the same time, adult trees are more adaptable to the environment and more actively uses nutrients than other stages (An & Shangguan, 2008). Ishida et al. (2005) found that water‐use efficiency was at its lowest in seedling leaves and that N content, net photosynthetic rate, and stomatal conductance were at their highest in sapling leaves, while in adult leaves, C:N ratio was at its highest and photosynthetic N‐use efficiency was at its lowest. Therefore, the nutrient utilization capacity of adult tree is stronger than that of sapling trees along altitudes environmental conditions.

## 5 CONCLUSIONS

Herein, we have introduced the life history stages of *A. mono* and detailed our study of the changes in leaf N‐P stoichiometry along an altitude gradient and further described the N‐P utilization strategy of *A. mono*. We concluded that N and P stoichiometric showed significant linear changed trend in different life history stages and at different altitudes. The N_mass_ and P_mass_ were greatly affected by environmental changes, while the N_area_ and P_area_ were greatly different by life history stage. *A. mono* leaf N‐P utilization strategy showed a significant allometric growth trend in all stages of life history as well as at low altitudes. The higher temperature and soil moisture conditions allowed *A. mono* to maintain a rapid growth state as an adult. Also, *A. mono* showed a significant allometric growth trend in all life history stages at medium altitudes. Finally, under different environmental conditions, the N‐P utilization strategies of *A. mono* at different stages of life history were found to be quite different. Our research suggests that future studies of plant responses to the environment should consider the influence of life history and provided the scientific basis for the study of plant nutrient utilization strategy on regional scale.

## CONFLICT OF INTEREST

The authors declare that they have no conflicts of interest.

## AUTHOR CONTRIBUTIONS

Z.S. analyzed the data and wrote the manuscript. Y.L. performed the experiments. J.H. designed the study. H.S. provided the experiments field and tool.

## Data Availability

The data used in this study are archived in the Dryad Data Repository (https://doi.org/10.5061/dryad.sbcc2fr2m).
