# Supplementary material for: N‐P utilization of Acer mono leaves at different life history stages across altitudinal gradients
Source: Ecol Evol. 2019 Dec 18;10(2):851–62. doi: 10.1002/ece3.5945 (PMC6988554; doi:10.1002/ece3.5945)
Supplement: Supplementary file 1 [file ECE3-10-851-s001.doc]

**Schedule table1.** Analysis of N-P content of *Acer mono* leaves under different environmental conditions and life history stages

|  |  | N_mass_(mg g^-1^) | P_mass_(mg g^-1^) | N_area_(mg cm^-2^) | P_area_(mg cm^-2^) | N:P |
| --- | --- | --- | --- | --- | --- | --- |
|  |  | ‾x±SD | ‾x±SD | ‾x±SD | ‾x±SD | ‾x±SD |
| Stage | Adult | 30.81±0.66b | 1.56±0.04 | 0.10±0.002c | 0.005±0.0001b | 20.02±0.43 |
|  | sapling | 30.61±0.54b | 1.61±0.07 | 0.09±0.002b | 0.005±0.0002b | 19.83±0.54 |
|  | Seedling | 27.91±0.58a | 1.51±0.05 | 0.07±0.002a | 0.004±0.0001a | 19.16±0.54 |
|  | F | 7.38** | 1.0 | 50.73** | 14.43** | 0.82 |
| T(°C) | 3 | 30.35±0.66b | 1.48±0.04a | 0.09±0.003a | 0.004±0.0001a | 21.08±0.67b |
|  | 3.6 | 25.69±0.51a | 1.40±0.03a | 0.09±0.003a | 0.005±0.0002b | 18.63±0.44a |
|  | 4.9 | 31.22±0.70b | 1.71±0.10b | 0.08±0.003a | 0.005±0.0003ab | 19.51±0.70ab |
|  | 5 | 31.85±0.58b | 1.66±0.04b | 0.10±0.003b | 0.005±0.0002b | 19.47±.0.41ab |
|  | F | 20.41** | 6.45** | 4.15** | 3.11* | 3.23* |
| SMC | 36.05 | 30.35±0.66b | 1.48±0.04a | 0.09±0.003a | 0.004±0.0001a | 21.08±0.67b |
| (%) | 13.39 | 25.69±0.51a | 1.40±0.03a | 0.09±0.003a | 0.005±0.0002b | 18.63±0.44a |
|  | 29.24 | 31.22±0.70b | 1.71±0.10b | 0.08±0.003a | 0.005±0.0003ab | 19.51±0.70ab |
|  | 51.29 | 31.85±0.58b | 1.66±0.04b | 0.10±0.003b | 0.005±0.0002b | 19.47±.0.41ab |
|  | F | 20.41** | 6.45** | 4.15** | 3.11* | 3.23* |

*Notes*: T is the abbreviation for temperature; SMC is the abbreviation of soil moisture content. Different lowercase letters in the same group represent significant differences between levels within the group. (**:*P*<0.01;*:*P*<0.05)
