# Supplementary material for: N‐P utilization of Acer mono leaves at different life history stages across altitudinal gradients
Source: Ecol Evol. 2019 Dec 18;10(2):851–62. doi: 10.1002/ece3.5945 (PMC6988554; doi:10.1002/ece3.5945)
Supplement: Supplementary file 2 [file ECE3-10-851-s002.doc]

**Schedule Table2.** Correlation analysis of N-P content and environmental change of *Acer mono* leaves

| Group | N_mass_ | P_mass_ | N_area_ | P_area_ | N:P |
| --- | --- | --- | --- | --- | --- |
| T(°C) | 18.93*** | 14.43*** | 3.15* | 5.33* | 1.91 |
| SMC(%) | 32.78*** | 1.33 | 4.43** | 0.20 | 4.91** |
| Stage | 15.69*** | 0.51 | 108.4*** | 28.04*** | 1.56 |
| T*SMC | 14.04*** | 3.4* | 12.84*** | 5.31* | 2.94* |
| T*Stage | _ | _ | _ | _ | _ |
| SMC*Stage | 0.3 | 0.69 | 0.09 | 1.62 | 2.44 |
| T*SMC *Stage | 0.02 | 0.02 | 6.80** | 1.88 | 0.13 |

*Notes*: T is the abbreviation for temperature; SMC is the abbreviation of soil moisture content. **P*<0.05;***P*<0.01; ****P*<0.001
