# Supplementary material for: N‐P utilization of Acer mono leaves at different life history stages across altitudinal gradients
Source: Ecol Evol. 2019 Dec 18;10(2):851–62. doi: 10.1002/ece3.5945 (PMC6988554; doi:10.1002/ece3.5945)
Supplement: Supplementary file 3 [file ECE3-10-851-s003.doc]

**Schedule Table 3.** Standardized Major Axis (SMA) regression parameters of leaf N-P content for each environment condition and life history stage.

|  | groups | n | P_mass_~N_mass_ | | | P_area_~N_area_ | | |
| --- | --- | --- | --- | --- | --- | --- | --- | --- |
|  |  |  |  | Slope(CI) | R^2^ |  | Slope(CI) | R^2^ |
| H | H1 | 45 |  | 0.76(0.59,0.98) | 0.34* |  | 0.89(0.73,1.08) | 0.59** |
|  | H2 | 45 |  | 0.49(0.39,0.62) | 0.40** |  | 0.62(0.51,0.75) | 0.60** |
|  | H3 | 45 |  | 0.96(0.72,1.28) | 0.10 |  | 1.02(0.85,1.24) | 0.61 |
|  | H4 | 45 |  | 0.75(0.56,1.00) | 0.05 |  | 0.87(0.70,1.08) | 0.48 |
| Stage | adult | 60 |  | 0.99(0.79,1.23) | 0.27 |  | 0.77(0.63,0.94) | 0.42* |
|  | young | 60 |  | 0.55(0.44,0.69) | 0.27** |  | 0.66(0.55,0.79) | 0.51** |
|  | seedling | 60 |  | 0.68(0.55,0.85) | 0.30** |  | 0.70(0.57,0.84) | 0.44** |

*Notes*: H1, H2, H3, and H4 represent abbreviations of the four altitude gradients respectively; H is the abbreviation for soil moisture content and temperature conditions; *: significant difference between slope and 1; **: extremely significant difference between slope and 1
