# Supplementary material for: N‐P utilization of Acer mono leaves at different life history stages across altitudinal gradients
Source: Ecol Evol. 2019 Dec 18;10(2):851–62. doi: 10.1002/ece3.5945 (PMC6988554; doi:10.1002/ece3.5945)
Supplement: Supplementary file 4 [file ECE3-10-851-s004.doc]

**Schedule Table 4.** Standardized Major Axis (SMA) regression parameters of leaf N-P stoichiometric traits of *Acer mono* for life history stages under different altitude

| groups | |  | N_mass_~P_mass_ | | | N_area_~P_area_ | | |
| --- | --- | --- | --- | --- | --- | --- | --- | --- |
| Factors | Stage | n | Slope(CI) |  | R^2^ | Slope(CI) |  | R^2^ |
| H1 | adult | 15 | 0.51(0.32,0.82) |  | 0.32** | 0.59(0.4,0.86) |  | 0.58** |
|  | young | 15 | 0.91(0.62,1.34) |  | 0.56 | 0.91(0.66,1.27) |  | 0.69 |
|  | seedling | 15 | 0.85(0.56,1.30) |  | 0.47 | 0.94(0.64,1.37) |  | 0.59 |
| H2 | adult | 15 | 0.51(0.3,0.87) |  | 0.14* | 0.66(0.45,0.97) |  | 0.57* |
|  | young | 15 | 0.45(0.31,0.65) |  | 0.59** | 0.54(0.41,0.71) |  | 0.79** |
|  | seedling | 15 | 0.43(0.26,0.70) |  | 0.25** | 0.55(0.35,0.86) |  | 0.38* |
| H3 | adult | 15 | 0.85(0.49,1.49) |  | 0.03 | 1.04(0.67,1.60) |  | 0.44 |
|  | young | 15 | 0.82(0.47,1.45) |  | 0.01 | 1.00(0.57,1.76) |  | 0.02 |
|  | seedling | 15 | 1.29(0.73,2.28) |  | 0.01 | 1.19(0.79,1.81) |  | 0.003 |
| H4 | adult | 15 | 1.12(0.67,1.88) |  | 0.20 | 0.7(0.43,1.12) |  | 0.33 |
|  | young | 15 | -0.45(-0.79,-0.26) |  | 0.004 | 0.88(0.51,1.52) |  | 0.08 |
|  | seedling | 15 | 0.69(0.40,1.149) |  | 0.09 | 0.45(0.28,0.74) |  | 0.29** |

*Notes*: H1, H2, H3, and H4 represent abbreviations of the four altitude gradients respectively; *: significant difference between slope and 1; **: extremely significant difference between slope and 1
